# Prophylaxe rezidivierender Harnwegsinfektionen mit Nitroxolin – Real-World-Daten aus der ProNitrox-Studie

**DOI:** 10.1007/s00120-025-02641-2

**Published:** 2025-07-28

**Authors:** A. Wiedemann, F. Wagenlehner, K. Naber, W. Strohmaier, W. Vahlensieck, R. Kirschner-Hermanns, A. Bannowsky, S. Wirz, J. Salem, T. Kuru

**Affiliations:** 1 Urologische Klinik am Ev. Krankenhaus Witten gGmbH, Lehrstuhl für Geriatrie, Universität Witten/Herdecke, Pferdebachstr. 27, 58455 Witten, Deutschland; 2 Klinik und Poliklinik für Urologie, Kinderurologie und Andrologie, Justus-Liebig-Universität, Gießen, Deutschland; 3 Abteilung für Urologie, Technische Universität München, München, Deutschland; 4 Sana Medical School Coburg, Universität Split, Split, Kroatien; 5 Urologie, Stresemannstraße 5, Bad Nauheim, Deutschland; 6 Neuro-Urologie, Neurologisches Rehabilitationszentrum Godeshöhe Bonn, Bonn, Deutschland; 7 Kliniken Landkreis Diepholz, Urologische Klinik, Diepholz, Deutschland; 8 Abteilung für Anästhesiologie, Intensivmedizin, Schmerz- und Palliativmedizin, Zentrum für Schmerzmedizin, Weaningzentrum, GFO-Kliniken Bonn/Cura Bad Honnef, Bad Honnef, Deutschland; 9 Klinik für Urologie und Kinderurologie, Universitätsklinikum Brandenburg a.d. Havel, MHB Brandenburg Theodor Fontane, Brandenburg a.d. Havel, Deutschland; 10 CUROS urologisches Zentrum, Klinik links vom Rhein, Köln, Deutschland

**Keywords:** Rezidivierende Zystitis, Langzeitprophylaxe, 8-Hydroxychinolinderivat, Antibiotika, Multimorbidität, Recurrent Cystitis, Long-term prophylaxis, 8-Hydroxyquinoline derivative, Antibiotics, Multimorbidity

## Abstract

**Hintergrund:**

Bei rezidivierenden Harnwegsinfektionen (rHWI) kann Nitroxolin, ein Hydroxychinolinderivat mit niedrigen Resistenzraten, zur Langzeitprophylaxe (LP) eingesetzt werden. Hierzu liegen bislang wenige Daten vor. Unter Real-World-Bedingungen sollten in einer retrospektiven, nicht-interventionellen Studie die klinische Routine einer LP mit Nitroxolin erhoben werden. Neben demografischen Daten war das Ausmaß der Multimorbidität, vorliegende Risikofaktoren für eine rHWI, die Art der Antibiotikatherapie der letzten akuten Episode der rHWI und die Quote der Durchbruchsinfektionen von besonderem Interesse.

**Material und Methoden:**

Im Zeitraum April bis Oktober 2024 wurden Daten von 360 Patienten zusammengetragen, die bei 70 Ärzten eine Reinfektionsprophylaxe mit Nitroxolin bei rHWI erhielten.

**Ergebnisse:**

Die Rate der Durchbruchsinfektionen lag bei 13 % und wurde überwiegend auf dem Boden einer Urinkultur ermittelt. Die dokumentierten Patienten waren zu 20 % (Frauen) bzw. 47 % (Männer) als multimorbid und zu 32 % (Frauen) bzw. 48 % (Männer) als geriatrisch einzustufen. 31,6 % der Patienten wiesen komplizierende Faktoren für eine rHWI auf. Die Therapiedauer mit Nitroxolin betrug überwiegend 3–6 Monate, in seltenen Fällen mehr als 2 Jahre.

**Schlussfolgerung:**

Die hohe Zahl von Komorbiditäten, höheres Alter, der hohe Anteil komplizierender Faktoren wie Harntraktanomalien und die Verwendung von Antibiotika in der letzten akuten Episode, die nicht für die LP der rHWI vorgesehen sind, charakterisieren die vorliegende Patientengruppe als schwierig. Dennoch ergibt sich eine günstige Quote von Durchbruchsinfektionen von 13 % unter der LP. Die am häufigsten genannten Gründe für die Auswahl von Nitroxolin für die LP waren gute Verträglichkeit und Wirksamkeit, die Resistenzlage sowie positive Erfahrungen des Therapeuten.

**Graphic abstract:**

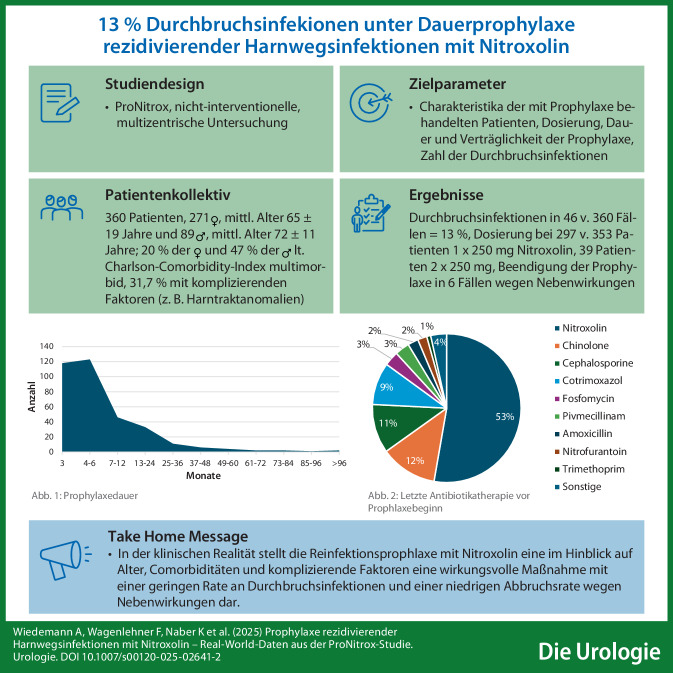

## Abstract (en)

**Background:**

For recurrent urinary tract infections (rUTI), nitroxoline, a hydroxyquinoline derivative with low resistance rates, can be used for long-term prophylaxis (LP). There is little data available on this so far. The clinical routine of LP with nitroxoline was to be assessed in a retrospective, noninterventional study under real-world conditions. In addition to demographic data, the extent of multimorbidity, existing risk factors for rUTI, the type of antibiotic therapy of the last acute episode of rUTI, and the rate of breakthrough infections were of particular interest.

**Materials and methods:**

In the period April–October 2024, data from 360 patients who received reinfection prophylaxis with nitroxoline for rUTI from 70 doctors were collected.

**Results:**

The rate of breakthrough infections was 13% and was predominantly determined from urine culture. Of the documented patients, 20% (women) and 47% (men) were classified as multimorbid and 32% (women) and 48% (men) were classified as geriatric. Furthermore, 31.6% of patients had complicating factors for rUTI. The duration of therapy with nitroxoline was usually 3–6 months, in rare cases more than 2 years.

**Conclusion:**

The high number of comorbidities, older age, the high proportion of complicating factors such as urinary tract abnormalities and the use of antibiotics in the last acute episode, which are not intended for LP of rUTI, characterize the present patient group as difficult. Nevertheless, there is a favorable recurrence rate of 13%. The most frequently cited reasons for choosing nitroxoline for LP were good tolerability and effectiveness, the level of resistance and the therapist’s positive experiences.

## Hinführung zum Thema

Harnwegsinfektionen (HWI) gehören zu den häufigsten Krankheitsbildern in der urologischen und hausärztlichen Sprechstunde. Zur Reinfektionsprophylaxe stehen leitliniengerecht antibiotische und nicht-antibiotische Therapien zur Verfügung. Unter den zur Verfügung stehenden Antibiotika in der Akuttherapie und der Reinfektionsprophylaxe nimmt Nitroxolin wegen seiner antibakteriellen Wirkung, die mit einer Hemmung der bakteriellen Adhäsion und der Degradierung von Biofilmen verknüpft ist, eine Sonderstellung ein. Daten zu einer Langzeitprophylaxe mit diesem Hydroxychinolinderivat sind bisher rar.

## Einleitung

In Zeiten zunehmender Antibiotikaresistenzen erleben „alte“ Antibiotika derzeit eine Renaissance. Unter diesen besitzt Nitroxolin, ein 8‑Hydroxychinolinderivat, durch seinen multimodalen Wirkansatz mit antibakteriellen [13], antimykotischen [7, 9] und sogar antitumoralen [16, 17, 27] Eigenschaften eine herausragende Stellung. In-vitro-Untersuchungen belegen einen präventiven Effekt gegen oxidativen Stress und deuten einen Einsatz bei neurodegenerativen Erkrankungen an [26]. Die antibakterielle Wirkung entsteht durch die Chelatbildung mit zweiwertigen Ionen wie Eisen oder Zink [21] und die Hemmung der Adhäsion von uropathogenen Keimen am Urothel [11]. Zusätzlich ist Nitroxolin ein Inhibitor von Metallo-β-Laktamasen [20] und kann Biofilme im Harntrakt degradieren [1, 23]. Es bestehen niedrige Resistenzraten gegen typische Erreger einer HWI wie Escherichia coli, aber auch eine Wirksamkeit gegen Problemkeime wie Acinetobacter baumannii [8] oder Aerococcus urinae [2] ist in vitro belegt. Für den seltenen Fall einer In-vitro-Resistenz gegen Nitroxolin ist eine Reduktion der „bakteriellen Fitness“ mit einem Verlust der Virulenz bzw. der Unfähigkeit, in vivo eine HWI auszulösen, nachgewiesen [5]. Ein neuer Literaturreview identifizierte 2 Arbeiten zu Resistenzraten von Nitroxolin, die für Nitroxolin mit 3,9 % von 1246 Enterobacterales bzw. 2 % von 394 MDR Enterobacterales aus Urinproben die jeweils niedrigsten Resistenzraten im Vergleich mit anderen getesteten Antibiotika fanden [12, 19, 24]. Dies korrespondiert mit 2 deutschen Untersuchungen, die für Nitroxolin bei der Therapie der akuten und rezidivierenden Zystitis in einer Dosierung von 3 × 250 mg über 5 bzw. 10 Tage klinische Heilungsraten von 90 bzw. 80 % angaben [18, 25]. Vor allem diese Daten haben dazu geführt, dass Nitroxolin in der S3-Leitlinie zur unkomplizierten HWI als Mittel der ersten Wahl neben Fosfomycin, Nitrofurantoin und Pivmecillinam bei unkomplizierten HWI in der Akuttherapie empfohlen wird [14, 15]. Bei rezidivierenden HWI (rHWI; ≥ 2/Halbjahr oder ≥3/Jahr) kann nach dem Versagen nicht-medikamentöser und nicht-antibiotischer medikamentöser Maßnahmen eine niedrig dosierte, antibiotische Langzeitprophylaxe (LP) durchgeführt werden [6]. Klinische Daten aus größeren Studien zu Erfahrungen mit Nitroxolin in der Langzeitprophylaxe von rHWI sind bisher rar [22]. Diese Lücke soll mit der vorliegenden nicht-interventionellen Studie „ProNitrox“ geschlossen werden.

## Material und Methoden

In der nicht-interventionellen Studie wurden mit einem Erhebungsbogen multizentrische Daten zu Patienten abgefragt, die Nitroxolin zur Langzeitprophylaxe rHWI erhalten haben. Hierzu gehörten neben demografischen Daten Informationen zu Häufigkeit und Art der HWI, Komorbiditäten, „urologische“ Risikofaktoren (wie z. B. Harntransportstörungen, angeborene Anomalien, Genitalsenkung der Frau etc.), antibiotische Vortherapien, Dosierung von Nitroxolin in der Langzeitprophylaxe, Prophylaxedauer, Entscheidungsgründe für die Auswahl von Nitroxolin, Nebenwirkungen und Gründe für die Beendigung der Langzeitprophylaxe. Daneben wurde die Anzahl der Durchbruchsinfektionen ermittelt und deren Diagnosegüte abgefragt. Ein besonderes Augenmerk wurde auf die Gruppe der „geriatrischen“ Patienten gelegt (definiert als ≥ 70 Jahre und multimorbid mit ≥ 2 Punkten im Charlson-Komorbiditätsindex (CCI; [4]) oder ≥ 80 Jahre), für die es ebenfalls bisher keinerlei Daten gab. Die Abfrage der Daten erfolgte durch das Online-Tool Survey Monkey Europe UC. Ein positives Ethikvotum (Antragsnummer: S‑60/2024) der Universität Witten-Herdecke lag vor. Die Studie wird beim BfArM unter der Studiennummer 7776 geführt.

## Ergebnisse

**Abb. 1:**
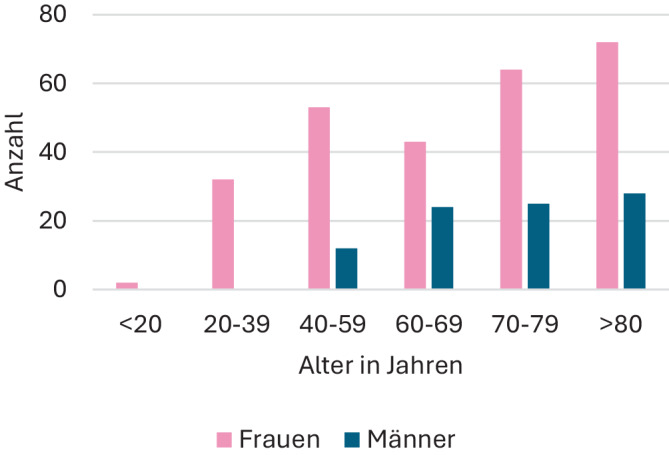
Altersverteilung von 360 eingeschlossenen Patienten (5 fehlende Altersangaben) der ProNitrox-Studie

Daten von 360 Patienten, die zu 98,6 % in urologischen Praxen behandelt wurden, flossen in die retrospektive Untersuchung ein. In allen Altersklassen dominierten Patientinnen, männliche Patienten traten erst ab der Altersgruppe der über 40-Jährigen auf. So lag das mittlere Alter der weiblichen Patienten (*n* = 271) mit 65 ± 19 Jahren unter dem der männlichen Patienten (*n* = 89) mit 72 ± 11 Jahren (s. Abb. 1).

Im CCI wiesen 54 von 271 Patientinnen (20 %) und 42 von 89 männlichen Patienten (47 %) einen Punktwert von ≥ 2 auf und firmierten als „multimorbid“. Der Anteil der Patienten mit Diabetes mellitus lag bei 19,2 % (*n* = 69, Männer: 25,8 %; Frauen 17 %). Insgesamt konnten 67 Frauen (23 %) und 39 Männer (48 %) durch die Kombination aus einem Alter ≥ 80 oder ≥ 70 Jahre und multimorbid mit einem CCI ≥ 2 Punkte als „geriatrisch“ adressiert werden.

**Abb. 2:**
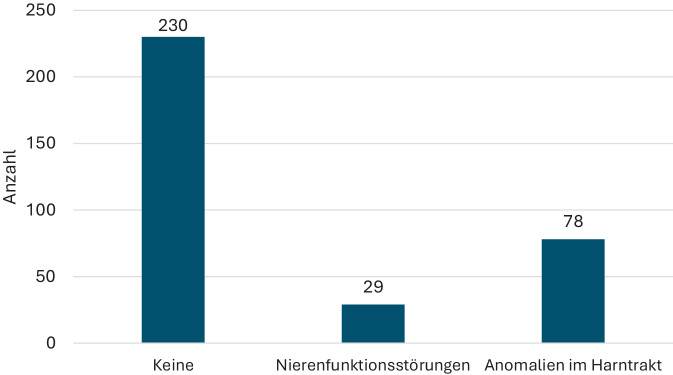
Dokumentierte komplizierende Faktoren (s. Text) bei Patienten der ProNitrox-Studie (23 ohne Angabe)

Bei 107 von 337 Patienten (31,7 %) wurden komplizierende Faktoren für eine HWI dokumentiert (s. Abb. 2). Zu den 78 Harntraktanomalien gehörten Nierenabgangsengen, Doppelnieren, vesikorenaler Reflux, Zystozele, Urolithiasis oder die Kombination aus mehreren Anomalien. Bei 29 Patienten lagen Nierenfunktionsstörungen vor.

**Abb. 3:**
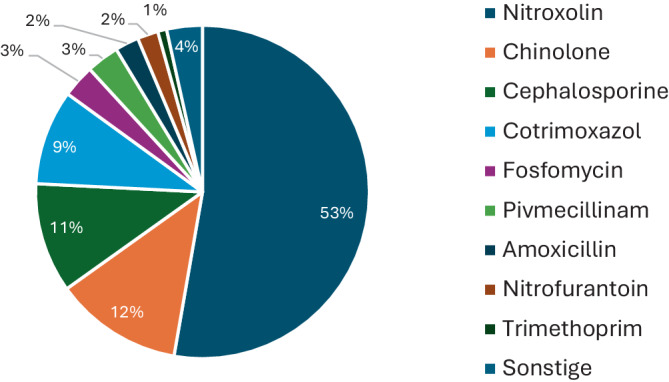
In der Primärtherapie der letzten akuten Harnwegsinfektion eingesetzte Antibiotika bei Patienten der ProNitrox-Studie (Sonstige: Piperacillin/Tazobactam, Ampicillin, Penicillin G, Meropenem, Ofloxacin)

Die Therapie der letzten akuten Zystitis bzw. des letzten Rezidivs vor Einleitung der Langzeitprophylaxe erfolgte mit den in Abb. 3 genannten Antibiotika.

**Abb. 4:**
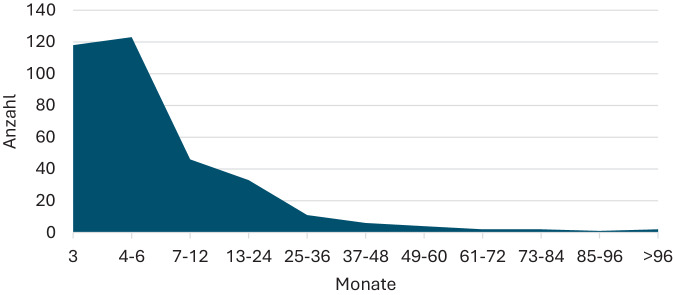
Dauer der Antibiotikaprophylaxe mit Nitroxolin in der ProNitrox-Studie (*n* = 348)

Es hatten 351 Patienten mit rHWI (98 %) eine leitliniengerechte Indikation einer Langzeitprophylaxe nach ≥ 2 symptomatischen Episoden innerhalb von 6 oder 3 oder mehr symptomatischen Episoden einer HWI innerhalb von 12 Monaten (*n* = 9: keine Angaben zur HWI-Häufigkeit). Die Mehrzahl der Patienten erhielt ihre Langzeitprophylaxe mit Nitroxolin über 3–6 Monate (s. Abb. 4). Vereinzelt wurde die Prophylaxe länger und bis zu 243 Monate kontinuierlich durchgeführt.

**Abb. 5:**
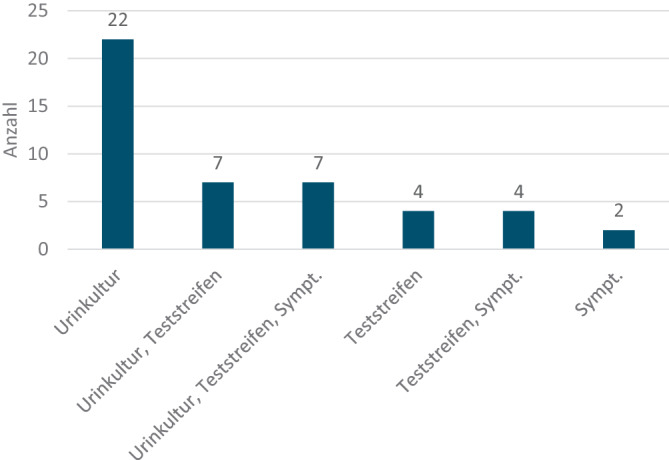
Zur Diagnose einer „Durchbruchsinfektion“ führende Tests in der ProNitrox-Studie

Eine Durchbruchsinfektion trat bei 46 von 360 Patienten (13 %) auf. Diese wurde in der Mehrzahl der Fälle mit Hilfe von Urinkulturen (48 %) oder Urinteststreifen oder Kombinationen aus beiden mit oder ohne Symptombeurteilung nachgewiesen bzw. rein klinisch (Abb. 5).

**Abb. 6:**
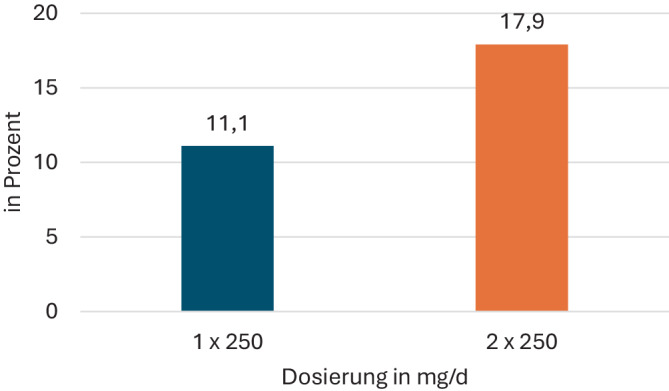
Häufigkeit der Durchbruchsinfektionen in Abhängigkeit von der Dosierung in der ProNitrox-Studie

Bei 297 von 353 Patienten (84,1 %) lag die Nitroxolin-Dosis bei einmal täglich 250 mg; 39 weitere Patienten (11,0 %) erhielten 2‑mal täglich 250 mg. In Einzelfällen (*n* = 14; 4 %) wurden andere Dosierungen gewählt (3 × 250 mg, 1 × 500 mg, 1 × 250 mg alle 2 Tage oder 2‑mal pro Woche bzw. 1 × 250 mg postkoital und nach 12 h). Unter der Dosierung von 2 × 250 mg pro Tag traten mehr Durchbruchsinfektionen auf als unter einer Dosierung von 1 × 250 mg (17,9 % vs. 11,1 %, Abb. 6). In der mit 2 × 250 mg Nitroxolin zur Langzeitprophylaxe behandelten Gruppe wiesen mehr Patienten komplizierende Faktoren auf (43,6 % vs. 28,4 %).

Bei 207 Patienten (57,5 %) wurde die Prophylaxe wie geplant ohne Durchbruchsinfektion beendet, bei 46 Patienten (12,8 %) war der Abbruchgrund eine Durchbruchsinfektion. In 16 Fällen (4,4 %) erfolgte das Absetzen auf Wunsch des Patienten, in weiteren 6 Fällen (1,7 %) wegen Nebenwirkungen und in 11 Fällen (3,1 %) wegen mangelnder Compliance (restliche Patienten – aktuell noch in andauernder Prophylaxe).

Gründe für die Auswahl von Nitroxolin für die Langzeitprophylaxe waren Verträglichkeit (292 Nennungen), Wirksamkeit (272 Nennungen), die Resistenzlage (220 Nennungen) und positive Erfahrungen der Therapeuten (218 Nennungen). Dahinter folgen weitere Entscheidungsgründe wie die Zulassung der Substanz zur Langzeitprophylaxe, Kontraindikationen bei Alternativen, Patientenwunsch bzw. Empfehlungen von Kollegen (81, 20, 20, 20 Nennungen). Als Kontraindikationen bei Alternativen, die zu einem Einsatz von Nitroxolin für die Langzeitprophylaxe führten, wurden gastrointestinale Beschwerden, Allergien und Niereninsuffizienz angeführt.

## Diskussion

In dieser multizentrischen, nicht-interventionellen Erhebung konnten erstmals Daten eines größeren Patientenkollektivs, das wegen rHWI eine Reinfektionsprophylaxe mit Nitroxolin erhielt, gesammelt werden. Während die Indikation zur Langzeitprophylaxe mit ≥ 2 Infektionsschüben in 6 bzw. ≥ 3 Schüben in 12 Monaten mit den Empfehlungen der Deutschen S3-Leitlinie „Epidemiologie, Diagnostik, Therapie, Prävention und Management unkomplizierter, bakterieller, ambulant erworbener Harnwegsinfektionen bei Erwachsenen“ übereinstimmt [6], zeigt die Prophylaxedauer in der klinischen Routine ein überraschendes Ergebnis: So wurden 17,5 % der Patienten über einen längeren Zeitraum als die üblichen 3–6 Monate einer Langzeitprophylaxe [15] mit Nitroxolin behandelt. Mit Ausnahme von 2 Patientinnen erhielten alle eine kontinuierliche Dauerprophylaxe mit 1 × 250 mg oder 2 × 250 mg Nitroxolin pro Tag, vereinzelt wurden auch andere Dosierungen gewählt. Die Möglichkeit einer symptomorientierten Therapie bei einem Rezidiv wurde nicht gewählt, die einer postkoitalen Einmaltherapie bei Bedarf in nur 2 Fällen. Hier erscheint eine Prophylaxedauer über einen Zeitraum von mehreren Jahren in Einzelfällen zumindest diskussionswürdig.

Hintergrund der z. T. langandauernden Rezidivprophylaxe könnte die Zahl der komplizierenden funktionellen oder anatomischen Faktoren darstellen. Diese fiel in der Gruppe der mit 1 × 250 mg täglich behandelten Patienten mit 28,4 % geringer aus als in der Gruppe der mit 2 × 250 mg Nitroxolin behandelten Patienten (43,6 %). Dies würde auch die höhere Anzahl von Durchbruchsinfektionen in der Gruppe der mit mehr als 1 × 250 mg prophylaktisch behandelten Patienten erklären. Hier führen offenbar nachvollziehbare komplizierende Faktoren etwa einer Nierenfunktionsstörung oder einer Harntraktanomalie wie dem vesikorenalen Reflux oder dem Pendelurin bei Doppelniere nicht nur zu einer höheren Prophylaxedosis, sondern auch zu einer erhöhten Quote von Durchbruchsinfektionen.

Auch die antibiotische Therapie der letzten zur Indikation einer Prophylaxe führenden HWI zeigte überraschende Ergebnisse: Da die deutsche S3-Leitlinie einen kritischen Einsatz dieser Antibiotikagruppen bei unkomplizierten Infektionen anmahnt, lässt die Verordnung von Chinolonen und Cephalosporinen in fast 23 % und in Einzelfällen sogar von Reserveantibiotika wie Piperacillin/Tazobactam oder Meropenem darauf schließen, dass es sich vor Einsatz der Prophylaxe um Infektionen mit resistenten Erregern gehandelt hat. Vor diesem Hintergrund – einen resistogrammgerechten Einsatz dieser Antibiotika bei den rezidivierenden Infekten angenommen – erscheint eine Quote von nur 13 % Durchbruchsinfektionen unter einer Prophylaxe mit Nitroxolin bei einer Patientengruppe, die in 47 % – entsprechend dem CCI – als multimorbid firmiert und bei der in 31,7 % komplizierende Harntraktanomalien nachgewiesen werden konnten, sehr gering. Da die Diagnose der Durchbruchsinfektion überwiegend durch eine Urinkultur bewiesen wurde und nur in Ausnahmefällen durch die Symptomatik oder einen Teststreifen allein, erscheint die Höhe von 13 % glaubhaft. Ein Vergleich mit anderen in der Langzeitprophylaxe einsetzbaren Antibiotika ist hier schwierig – Studien hierzu sind teilweise 20 Jahre alt und im Hinblick auf die Patientencharakteristika, das Keimspektrum und die Therapiedauer nicht unbedingt vergleichbar [10]. In einer Metaanalyse fand sich bei der Langzeitprophylaxe nicht schwangerer Frauen mit rHWI eine Rezidivrate von 15 % [3]. Insbesondere die Komorbiditäten wurden in anderen Untersuchungen häufig nicht strukturiert erfasst.

Die Zahl von 13 % Durchbruchsinfektionen erscheint auch vor dem Hintergrund des hohen Anteils geriatrischer Patienten niedrig: So erfüllten 23 % der Frauen und 48 % der Männer die Definition eines geriatrischen Patienten durch die Kombination aus Alter und Komorbiditäten. Dies ist vor dem Hintergrund der damit verbundenen Polypharmazie und den Limitationen durch die Komorbiditäten selbst (sinkende GFR, sinkende Leberleistung, steigende Prävalenz eines Diabetes mellitus etc.) erstaunlich. Offenbar wird in der ärztlichen Routine nicht nur die junge, prämenopausale Frau mit rHWI, sondern auch der multimorbide geriatrische Patient mit einer Dauerprophylaxe mit Nitroxolin behandelt. Bemerkenswert ist in diesem Zusammenhang auch, dass Nitroxolin gerade wegen seiner Verträglichkeit für dieses Patientenklientel ausgewählt wurde. Diese wird durch die z. T. lange Prophylaxedauer und durch die geringe Anzahl von Patienten, die auf eigenen Wunsch ein Absetzen verlangten, indirekt bestätigt.

## Schlussfolgerungen

Erstmals wurden therapiebegleitend Daten von 360 Patienten, die Nitroxolin als Langzeitprophylaxe von rezidivierenden Harnwegsinfektionen (rHWI) erhielten, zusammengetragen. Die Indikation für eine Langzeitprophylaxe wurde ganz überwiegend leitliniengerecht gemäß der Definition einer rHWI gestellt. Diese Patientengruppe stellte sich mit einem mittleren Alter der Frauen von 65 ± 19 Jahren und der Männer von 72 ± 11 Jahren, einem Anteil von multimorbiden Patienten von 47 % – gemessen mit dem Charlson-Komorbiditätsindex (CCI), einer Quote von 31,6 % komplizierender harntraktbezogener Risikofaktoren und einem Anteil von 19,2 % als älter und multimorbider dar als die klassischerweise prophylaktisch behandelte Patientengruppe der jüngeren, prämenopausalen Frau. Die antibiotische Therapie der letzten akuten Episode der HWI vor Einleitung der Langzeitantibiose deutet mit einem fast 23 %igen Anteil von Chinolonen oder Cephalosporinen als nicht für die Primärtherapie einer unkomplizierten Zystitis in der deutschen S3-Leitlinie gelisteten Substanz auf das Vorliegen einer Urinbakteriologie mit eher resistentem Keimspektrum hin. Vor diesen Hintergründen ist eine überwiegend per Uricult gestellte Diagnose einer Durchbruchsinfektion mit 13 % aller Patienten als ungewöhnlich niedrig zu bewerten. Die Häufigkeit der Durchbruchsinfektionen unterschied sich nach dem gewählten Dosierungsregime – sie war bei den mit mehr als 1 × 250 mg Nitroxolin behandelten Patienten höher. In dieser Gruppe lag auch der Anteil komplizierender Harntraktanomalien höher als in der Gruppe der mit 1 × 250 mg behandelten Patienten. Die Dauer der prophylaktischen Gabe von Nitroxolin lag überwiegend bei 3–6 Monaten, in Einzelfällen erreichte sie sogar eine Dauer von über 2 Jahren. Zur Beendigung der Dauerprophylaxe führte überwiegend das geplante Ende der Prophylaxephase; Nebenwirkungen, eine mangelnde Compliance oder der Patientenwunsch spielten als Absetzgrund eine untergeordnete Rolle. Die Quote von nur 6 ungeplanten Abbrüchen der Langzeitprophylaxe mit Nitroxolin wegen Nebenwirkungen deutet auf eine gute Verträglichkeit der Substanz auch in der Langzeitgabe hin. Zusammenfassend erscheint eine Langzeitprophylaxe mit Nitroxolin auch bei rezidivierenden komplizierten HWI eines überwiegend älteren und multimorbiden Patientenklientels eine effektive und nebenwirkungsarme Option. Verbesserungspotentiale ergeben sich u. U. in dem Ausnutzen flankierender Maßnahmen wie einer lokalen Östrogenisierung der postmenopausalen Frau oder der Gabe von Nitroxolin als postkoitale Einmalprophylaxe respektive symptomgetriggerte Eigentherapie anstelle einer langfristigen Dauergabe bzw. der Beseitigung komplizierender Faktoren im Harntrakt durch entsprechende urologische Maßnahmen.

## Fazit für die Praxis

Eine Harnwegsinfektionsprophylaxe mit Nitroxolin führt in der vorliegenden nicht-interventionellen Untersuchung lediglich zu einer nur geringen Rezidivquote von 13 % Durchbruchsinfektionen bei einem häufig älteren und multimorbiden Patientenklientel mit einem hohen Anteil von Risikofaktoren für rezidivierende Harnwegsinfektionen. Die Langzeitprophylaxe ist auch über viele Monate mit einer geringen Nebenwirkungsrate durchführbar. Deswegen sollte, wenn nicht-antibiotische Prophylaxemaßnahmen versagt haben, an eine antibiotische Langzeitprophylaxe mit Nitroxolin, das wegen seiner über Jahre exzellenten Resistenzraten häufig in Resistogrammen erst gar nicht getestet wird, bei rHWI vermehrt gedacht werden.

## Data Availability

Die erhobenen Datensätze können auf begründete Anfrage in anonymisierter Form beim korrespondierenden Autor angefordert werden. Die Daten befinden sich auf einem Datenspeicher bei der kyoups GmbH, Alfeld (Leine).

## References

[1] Abouelhassan Y, Yang Q, Yousafd H, Nguyen MT, Rolfe M, Schultz GS, Huigens RW (2017) Nitroxoline: a broad-spectrum biofilm-eradicating agent against pathogenic bacteria. Int J Antimicrob Agents 49:247–251 28110918 10.1016/j.ijantimicag.2016.10.017

[2] Ahmadzada A, Fuchs F, Hamprecht A (2023) Susceptibility of Aerococcus urinae and Aerococcus sanguinicola to Standard Antibiotics and to Nitroxoline. Microbiol Spectr 11:10.1128/spectrum.02763-22 PMC10100651 36847493

[3] Albert X, Huertas I, Pereiro I, Sanfélix J, Gosalbes V, Perrotta C (2004) Antibiotics for preventing recurrent urinary tract infection in non-pregnant women. Cochrane Database Syst Rev. 10.1002/14651858.CD001209.pub2 15266443 10.1002/14651858.CD001209.pub2 PMC7032641

[4] Charlson ME, Pompei P, Ales KL, MacKenzie CR (1987) A new method of classifying prognostic comorbidity in longitudinal studies: development and validation. J Chronic Dis 40:373–383 3558716 10.1016/0021-9681(87)90171-8

[5] Deschner F, Risch T, Baier C, Schluter D, Herrmann J, Muller R (2024) Nitroxoline resistance is associated with significant fitness loss and diminishes in vivo virulence of Escherichia coli. Microbiol Spectr 12:e307923 38063385 10.1128/spectrum.03079-23 PMC10782962

[6] Deutsche Gesellschaft für Urologie e. V (2024) https://register.awmf.org/de/leitlinien/detail/043-044. Zugegriffen: 18. Jan. 2025

[7] Fuchs F, Aldejohann AM, Hoffmann AM, Walther G, Kurzai O, Hamprecht AG (2022) In Vitro Activity of Nitroxoline in Antifungal-Resistant Candida Species Isolated from the Urinary Tract. Antimicrob Agents Chemother 66:10.1128/aac.02265-21 PMC9211423 35543103

[8] Fuchs F, Becerra-Aparicio F, Xanthopoulou K, Seifert H, Higgins PG (2022) In vitro activity of nitroxoline against carbapenem-resistant Acinetobacter baumannii isolated from the urinary tract. J Antimicrob Chemother 77:1912–1915 35411393 10.1093/jac/dkac123

[9] Fuchs F, Hof H, Hofmann S, Kurzai O, Meis JF, Hamprecht A (2021) Antifungal activity of nitroxoline against Candida auris isolates. Clin Microbiol Infect 27(1697):e7–e10 10.1016/j.cmi.2021.06.035 34245904

[10] Hooton TM (2001) Recurrent urinary tract infecton in women. Int J Antimicrob Agents 17:259 11295405 10.1016/s0924-8579(00)00350-2

[11] Karam D, Amgar A, Bourlioux P (1988) Inhibition of bacterial adhesion of uropathogenic Escherichia coli strains by the urine of patients treated with nitroxoline. Pathol Biol (paris) 36:452–455 3043342

[12] Krajewski W (2024) Nitroxoline: treatment and prevention of urinary tract infections from the urologist’s perspective. Cent European J Urol 77:339–343 39345309 10.5173/ceju.2024.17 PMC11428350

[13] Kresken M, Korber-Irrgang B (2014) In vitro activity of nitroxoline against Escherichia coli urine isolates from outpatient departments in Germany. Antimicrob Agents Chemother 58:7019–7020 25182654 10.1128/AAC.03946-14 PMC4249399

[14] Kranz J, Helbig S, Mandraka F, Schmidt S, Naber KG (2017) The revival of old antibiotics for treatment of uncomplicated urinary tract infections in the era of antibiotic stewardship. Curr Opin Urol 27:127–132 27875349 10.1097/MOU.0000000000000365

[15] Kranz J et al (2018) The 2017 Update of the German Clinical Guideline on Epidemiology, Diagnostics, Therapy, Prevention, and Management of Uncomplicated Urinary Tract Infections in Adult Patients. Part II: Therapy and Prevention. Urol Int 100(3):271–278 29539622 10.1159/000487645

[16] Lazovic JJ, Guo L, Nakashima J, Mirsadrei L, Yong W, Kim HJ, Ellingson B, Wu H, Pope WB (2015) Nitroxoline induces apoptosis and slows glioma growth in vivo. Neuro Oncol 17:53–62 25074541 10.1093/neuonc/nou139 PMC4483047

[17] Mitrovic A, Kos J (2019) Nitroxoline: repurposing its antimicrobial to antitumor application. Acta Biochim Pol 66:521–531 31834689 10.18388/abp.2019_2904

[18] Naber KG, Niggemann H, Stein G, Stein G (2014) Review of the literature and individual patients’ data meta-analysis on efficacy and tolerance of nitroxoline in the treatment of uncomplicated urinary tract infections. BMC Infect Dis 14:628 25427651 10.1186/s12879-014-0628-7 PMC4262220

[19] Plambeck L, Fuchs F, Sattler J, Hamprecht A (2022) In vitro activity of mecillinam, temocillin and nitroxoline against MDR. Enterobacterales Jac Antimicrob Resist 2022(4):dlac59 10.1093/jacamr/dlac059 PMC9201239 35719201

[20] Proschak A et al (2022) Nitroxoline and its derivatives are potent inhibitors of metallo-beta-lactamases. Eur J Med Chem 228:113975 34865870 10.1016/j.ejmech.2021.113975

[21] Repac Antic D, Parcina M, Gobin I, Petkovic Didovic M (2022) Chelation in Antibacterial Drugs: From Nitroxoline to Cefiderocol and Beyond. Antibiot (basel) 11(8):1005 10.3390/antibiotics11081105 PMC9405089 36009974

[22] Sachse D (1984) Therapie chronisch-rezidivierender Harnwegsinfekte mit Nitroxolin. Therapiewoche 34(68):228–230

[23] Klinger SAM (2012) The urinary antibiotic 5‑nitro-8-hydroxyquinoline (Nitroxoline) reduces the formation and induces the dispersal of Pseudomonas aeruginosa biofilms by chelation of iron and zinc. Antimicrob Agents Chemother 56:6021–6025 22926564 10.1128/AAC.01484-12 PMC3486607

[24] Stoltidis-Claus C, Rosenberger KD, Mandraka F et al (2023) Antimicrobial resistance of clinical Enterobacterales isolates from urine samples, Germany, 2016 to 2021. Euro Surveill 28:2200568 37166759 10.2807/1560-7917.ES.2023.28.19.2200568 PMC10176829

[25] Wagenlehner F, Kresken M, Wohlfahrt E, Bahrs C, Grabein B, Strohmaier WL, Naber KG (2023) Therapie der Zystitis mit Nitroxolin – NitroxWin. Prospektive, multizentrische, nicht-interventionelle Studie und mikrobiologische Untersuchungen zur Resistenzsituation. Urologie 62:1186–1192 37650911 10.1007/s00120-023-02167-5 PMC10630225

[26] Van Hau T, Ruankham W, Suwanjang W, Songtawee N, Wongchitrat P, Pingaew R, Prachayasittikul V, Prachayasittikul S, Phopin K (2019) Repurposing of Nitroxoline Drug for the Prevention of Neurodegeneration. Chem Res Toxicol 32:2182–2191 31638783 10.1021/acs.chemrestox.9b00183

[27] Xu N, Lin W, Sun J, Sadahira T, Xu A, Watanabe M, Guo K, Araki M, Li G, Liu C, Nasu Y, Huang P (2020) Nitroxoline inhibits bladder cancer progression by reversing EMT process and enhancing anti-tumor immunity. J Cancer 11:6633–6641 33046984 10.7150/jca.47025 PMC7545671

